# Ultrasound-Assisted Extraction of Phenolics from Pear Pomace: Method Optimization, Phenolic Profile, and Antioxidant Capacity

**DOI:** 10.3390/molecules31111938

**Published:** 2026-06-03

**Authors:** Violeta Nour, Alexandra Cicanci, Alexandru Radu Corbu, Iurie Rumeus, Liliana Ceclu

**Affiliations:** 1 Department of Horticulture & Food Science, University of Craiova, 13 AI Cuza Street, 200585 Craiova, Romania; corbu_lx@yahoo.co.uk; 2 Faculty of Economics, Engineering and Applied Sciences, Cahul State University “B.P. Hasdeu”, Piaţa Independenţei, 1, 3900 Cahul, Moldova; alexandra.cicanci@feisa.usch.md (A.C.); iurie.rumeus@feisa.usch.md (I.R.); liliana.ceclu@feisa.usch.md (L.C.)

**Keywords:** pear pomace, ultrasound-assisted extraction, response surface methodology, ultrasonic power, ethanol concentration, extraction time, phenolic compounds

## Abstract

Pear pomace, a major by-product of the juice processing, represents a valuable yet underexploited source of bioactive compounds. In this study, ultrasound-assisted extraction (UAE) was optimized using response surface methodology to enhance the recovery of phenolic compounds from pear pomace powder with aqueous ethanol as the extraction solvent. A Box–Behnken design was employed to evaluate the effects of ethanol concentration (40–80%), extraction time (30–90 min), and ultrasonic power (48–144 W) on total phenolic content and DPPH radical scavenging activity. The results demonstrated that the negative quadratic effect of ultrasonic power on phenolic extraction yield exhibited the highest level of statistical significance. Extraction using 40% ethanol for 64.45 min at a power intensity of 117.6 W enabled the optimal recovery of phenolic constituents, yielding 280.1 mg GAE/L in the extracts. Using HPLC, the study explored how variations in ethanol concentration and extraction time affect the phenolic composition of the extracts. Chlorogenic acid, rutin and epicatechin were found to be the major polyphenolic compounds. Overall, pear pomace demonstrated strong potential as a low-cost source of phenolic compounds, and the optimized UAE process proved to be an efficient and robust approach for maximizing phenolic recovery.

## 1. Introduction

Pears, which are classified within the genus *Pyrus* of the Rosaceae family (subfamily Pyrinae), represent a fruit crop of considerable economic significance on a global scale [1,2]. Globally, pear production is second only to apple production, highlighting its significant importance among major fruit crops. In 2024 alone, about 27.63 million tons of pears were harvested across more than 90 producing countries, of which 83.3% originated from Asia and 8.4% from European countries [3]. Owing to their delicious taste and attractive aromas, pears are widely enjoyed around the world, both as fresh fruit and in a variety of processed products, including juices, syrups, confectionery, preserved fruits, jams and ice creams [4,5]. Moreover, pear consumption has been consistently linked to wide range of health benefits, including antioxidant, anti-inflammatory, and antiproliferative activities, as extensively documented in the scientific literature [6,7].

The extraction of pear juice by pressing yields up to 35% pomace—a nutritionally rich by-product comprising peels, pulps, stems, cores, and seeds that presents significant challenges in terms of waste management, as well as economic and environmental concerns [1,8]. Only about 20% of pomace is effectively utilized, mainly as a supplement in livestock nutrition, organic fertilizers, or as a source of pectin for ethanol production, with the remainder being discarded as waste [9,10]. The improper management of these by-products leads to depletion of essential nutrients and beneficial bioactive compounds, especially in contexts where malnutrition remains a concern [11,12]. Reintegrating fruit pomace into the food value chain represents a more sustainable and environmentally responsible approach, while also enhancing the overall efficiency of the fruit processing industry. The market for fruit pomace valorization is forecasted to grow from $3.4 billion in 2025 to $4.65 billion by 2033, driven by rising consumer awareness of sustainability and health-conscious eating, alongside increasing demand for natural and organic food products [13]. Owing to its high moisture content (60–70%), pear pomace is highly perishable. Consequently, moisture reduction techniques—particularly drying—must be employed to preserve its physical and chemical properties, prevent spoilage, and reduce transportation costs [14].

Pear pomace represents a promising source of bioactive compounds, especially polyphenols and triterpenes [15], which function as powerful antioxidants and free-radical scavengers and are linked to a broad spectrum of health benefits, including antidiabetic, antitumor, anti-inflammatory, anti-aging, antihyperglycemic, cardioprotective, and neuroprotective effects [8,16,17]. In addition, pear pomace also serves as a potential source of other valuable ingredients, including dietary fiber, vitamins, and minerals [8].

Previous chemical analyses have shown that the major phenolic compounds identified in both pear peel and pulp include chlorogenic, caffeic, and coumaric acids, as well as epicatechin and rutin, in addition to triterpenes such as oleanolic and ursolic acids, procyanidins and arbutin [18,19]. The results indicated markedly higher concentrations in the peel compared to the pulp, highlighting that substantial amounts of bioactive compounds are lost in pear pomace at different stages of pear processing [15,20,21]. Phenolic compounds represent an important target in the valorization of fruit by-products due to their wide range of bioactive properties, as well as their functional and technological applications as natural additives and health-promoting food ingredients [22].

At present, there is a rising focus on green extraction methods aimed at the recovery of bioactive compounds from fruit processing by-products [23]. Compared with the ‘traditional’ extraction procedures, such as hydro-distillation, Soxhlet extraction or conventional maceration, ultrasound-assisted extraction (UAE) is regarded as an innovative and environmentally sustainable method, with strong potential to minimize environmental impact by reducing energy demand and solvent usage and by enabling the use of safer solvents, while simultaneously enhancing the extraction efficiency and the quality of the obtained compounds [24,25]. UAE operates through acoustic cavitation induced by high-intensity ultrasonic waves, which disrupt cellular structures and enhance mass transfer, leading to improved extraction efficiency and significantly reduced processing times [26]. To achieve maximum efficiency, the extraction conditions must be carefully optimized for each specific plant material. Response surface methodology (RSM) integrated with factorial experimental designs is a well-established and effective statistical modeling approach used for optimizing processes involving multiple responses [27].

Few studies have so far focused on the ultrasound-assisted recovery of phenolic compounds from pear pomace [1,8]. Therefore, this study aimed to design and optimize an efficient ultrasound-assisted extraction process using aqueous-ethanolic solvents for the green recovery of phenolic compounds from pear pomace. The Box–Behnken design was applied to determine the optimal extraction parameters—ethanol concentration, extraction time, and ultrasonic power—in order to maximize the yield of phenolic compounds and enhance the radical scavenging activity of pear pomace extracts. Additionally, the extracts were characterized in terms of their individual phenolic compounds using high-performance liquid chromatography with diode-array detection (HPLC-DAD).

## 2. Results and Discussion

### 2.1. Compositional Characterization of Pear Pomace Powder

Table 1 presents the proximate of pear pomace powder. The results indicate 3.84%, 0.77% and 1.52% for protein, fat and ash content, respectively. Krajewska and Dziki [28] found lower protein (2.64%) but higher fat (0.92%) and ash (1.99%) content in freeze-dried pear pomace, and Rocha-Parra et al. [29] reported 4.78% protein, 1.90% fat and 1.06% ash content in pear pomace dried in a forced-convection oven while Kausar et al. [30] reported 0.95% fat and 2.95% protein in pear pomace dried in a hot air oven at 60 °C. Bozdogan et al. [31] determined the protein, oil, and ash contents of the pear pomace powder as 2.89%, 1.88%, and 1.20%, respectively. A substantial total phenolic content (297.98 mg/100 g) was observed in pear pomace powder, in agreement with earlier findings.

Krajewska et al. [10] found that TPC in pear pomace was influenced by the drying method and temperature, ranging from 258 to 432 mg GAE/100 g, while Ferreira et al. [1] determined a slightly higher content in pear pomace powder obtained by convective air-drying at 80–85 °C, ranging from 376 to 513 mg GAE/100 g, depending on particle size and extraction method. In line with the present results, Jiang et al. [32] found a total phenolic content of 261.96 mg GAE/100 g in Asian pear powder.

The content of individual phenolic compounds in pear pomace powders, as quantified by HPLC-DAD, is shown in Table 2.

The most abundant were chlorogenic acid (26.41 mg/100 g) and rutin (18.32 mg/100 g), followed by epicatechin (6.26 mg/100 g) and vanillic acid (3.61 mg/100 g). Consistent with these findings, Krajewska et al. [10] found chlorogenic acid as one of the dominant polyphenolic acids in pear pomace (29 mg/100 g), but reported much higher levels of rutin, ranging from 92 to 267 mg/100 g. Beyond chlorogenic acid and rutin, they quantified multiple phenolic compounds in pear pomace extracts, including isoquercitrin, epicatechin, caffeic acid, gallic acid and quercetin. Jiang et al. [32] also reported chlorogenic acid as one of the most abundant phenolic compounds in pear pomace powder. They quantified 9.56 and 8.84 mg/100 g, but higher content of coumaric acid (20.01 and 18.68 mg/100 g) in freeze-dried and hot air-dried Asian pear powder, respectively. The differences could be attributed to variations in fruit composition, determined by variety, geographic origin, environmental factors, stage of ripening of the fruits, and in fruit processing technologies. Differences in drying conditions and analytical methodologies may also account for the observed variations in the composition of the pomace powders.

### 2.2. Box–Behnken Design Results

In this study, UAE of pear pomace powder in aqueous ethanol was optimized using RSM to maximize total phenolic content (TPC) and DPPH radical scavenging activity (RSA) in the extracts. A Box–Behnken design comprising 15 experimental runs performed in duplicate was used to optimize the extraction conditions, namely the ethanol concentration, ultrasonic power and extraction time. Table 3 presents the observed and predicted values of TPC and RSA of pear pomace extracts, with the predicted values generated using the fitted model.

A comparison between the predicted values and the experimental results showed close agreement across all evaluated extraction conditions. The observed values deviated from the predicted ones by no more than 5%. The TPC and RSA of pear pomace extract ranged from 156.55 to 263 mg GAE/L and from 0.44 to 0.89 mmol Trolox/L, respectively.

Table 4 displays the coefficients of the quadratic models predicting TPC and RSA, along with the associated ANOVA results for each model. It provides essential statistical information for assessing model fit and the significance of individual terms. In addition, these findings provide insight into the manner in which the independent variables affect the responses, whereby positive coefficient values indicate synergistic interactions, while negative values reflect antagonistic behavior.

The ANOVA table decomposes the total variability in total phenolic content into distinct components attributable to each effect. The statistical significance of these effects is then evaluated by comparing their mean square values with an estimate of the experimental error. The models demonstrated strong fits, with R-squared values of 97.66% for TPC and 92.42% for RSA, indicating that a high proportion of the variability in the responses was explained. Moreover, the regression tests returned *p*-values below 0.05, providing strong evidence that both models accurately and reliably describe the relationships among the variables. Mathematical modeling allows for the generation of response surfaces for each response variable under investigation.

### 2.3. Effect of UAE on Total Phenolic Content

**Figure 1:**
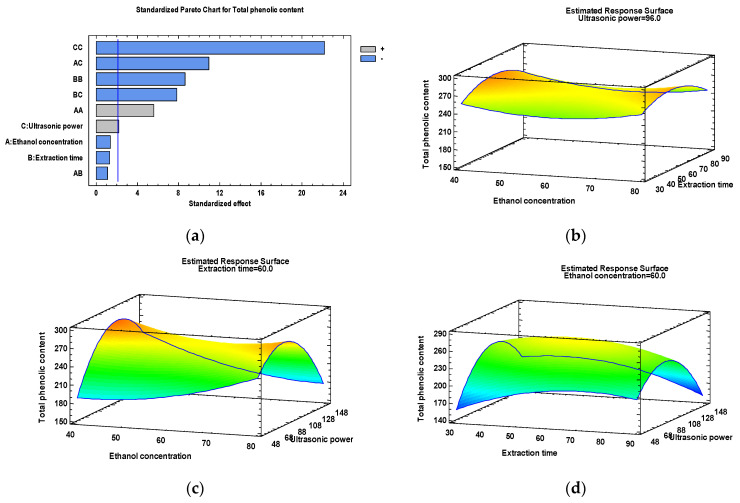
Pareto chart (**a**) and estimated response surface graphs of TPC (mg GAE/L) as a function of ethanol concentration and extraction time at 96 W ultrasonic power (**b**), ethanol concentration and ultrasonic power at 60 min extraction time (**c**) and extraction time and ultrasonic power at 60% ethanol concentration (**d**).

The optimal combination of process parameters for maximizing TPC was found to be 40% ethanol concentration, an extraction time of 65.45 min, and an ultrasonic power of 117.7 W. The model predicted a maximum TPC of 280.1 mg GAE/L under the optimized conditions. As depicted in the Pareto chart (Figure 1a), the three quadratic terms, the linear term and the two interaction terms of ultrasonic power show *p*-values below 0.05, indicating that they are statistically significant at the 95% confidence level. Moreover, the Pareto plot shows that the quadratic effect of ultrasonic power was the most significant, followed by the interaction effect of ethanol concentration and ultrasonic power. The negative quadratic effect of ultrasonic power on phenolic extraction yield indicated that there is a maximum for the extraction yield of phenolic compounds at a certain ultrasonic power. González-Centeno et al. [33] also reported that both ultrasonic frequency and power had significant effect (*p* < 0.05) on the aqueous UAE of grape pomace. Figure 1b–d presents the three-dimensional response surface plots showing the effect of ethanol concentration, extraction time and ultrasonic power on extraction of phenolic compounds from pear pomace.

Both the quadratic model coefficients and the Pareto chart revealed a positive contribution of ultrasonic power to TPC. As presented in Table 3, when increasing the ultrasonic power from 48 W to 144 W, TPC in the extract increased by 34.65% (from 156.54 mg/L to 210.63 mg/L) after 30 min extraction time in 60% ethanol.

Van Man et al. [34] investigated UAE of phenolic compounds from mashed tea leaves and reported a 16.6% increase in yield when power intensity was raised from 25 to 125 W. Maran et al. [35] and Da Porto et al. [36] similarly reported increased polyphenol yields in *Nephelium lappaceum* fruit peel extract and grape seed extract, respectively, when ultrasonic power was raised from 50 to 150 W.

The cavitation generated by ultrasonic waves is widely recognized for producing cavitation bubbles, whose formation and collapse facilitate interparticle collisions and the structural breakdown of cellular matrices [37,38], thereby promoting more effective solvent permeation within the matrix and accelerating the mass transfer of phenolic compounds [39,40]. The occurrence of acoustic cavitation is strongly influenced by the pressure amplitude of the sound wave and, consequently, by the ultrasonic intensity. However, the efficiency of acoustic cavitation depends also on the solvent’s physicochemical properties and the way these properties interact with the ultrasound [41]. The highest TPC yield was achieved at approximately 120 W and decreased at higher ultrasonic power levels, as shown in Figure 1d when using 60% ethanol as the solvent. Some previous studies have also reported that using UAE at high ultrasonic powers may have detrimental effects, including the degradation of phenolic compounds and some other bioactive compounds [42]. The degradation of specific phenolic compounds induced by UAE, particularly at high ultrasonic power, is regarded as one of its main disadvantages [43]. This effect can be explained by the enhanced formation of hydroxyl radicals at higher ultrasound powers, which promote the degradation of polyphenols, particularly in systems with high water content [44].

The stability of polyphenols following UAE-induced cavitation is influenced by the chemical nature of the phenolic compound [38]. Belwal et al. [45] reported the complete degradation of caffeic, chlorogenic, p-coumaric, and 4-hydroxybenzoic acids in *Berberis jaeschkeana* C.K. Schneid. fruits following UAE, while Celotti et al. [46] reported that higher ultrasound amplitude resulted in reduced extraction yields of flavan-3-ols in red young wines due to chemical degradation. However, according to Pingret et al. [47], epicatechin and chlorogenic acid—the main polyphenols in apple pomace—showed stability during ultrasound-assisted aqueous extraction.

After removal the non-significant factors (*p* > 0.05), the following reduced predictive equation could describe the polynomial model for total phenolic content:

(1) $$\begin{array}{cc} TPC=\;-189.852 & -1.65869\times x_{1}+4.41714\times x_{2}+7.60919\times x_{3}+0.037213\times x_{1}^{2}\\ & -0.0292383\times x_{1}x_{3}-0.0256345\times x_{2}^{2}-0.0139688\times x_{2}x_{3}\\ & -0.0256976\times x_{3}^{2} \end{array}$$

**Figure 2:**
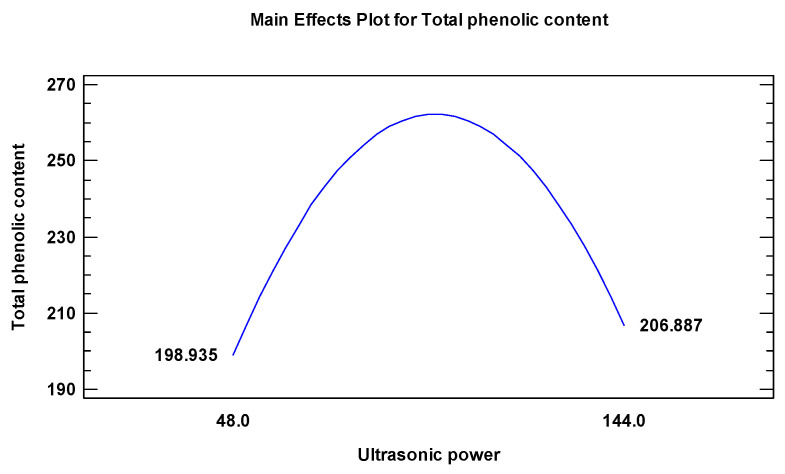
Main effects plot for total phenolic content in the reduced model.

The main effects plot of this model is presented in Figure 2. The R-squared of this model was 97.0661%. Optimum TPC value (281.68 mg GAE/L of extract) is obtained under extraction with 40% ethanol during 56.34 min at an ultrasonic power of 110.27 W.

Ethanol is an effective solvent for the extraction of polyphenols, and, in addition to its recognized safety for food applications, it is also valued for its green character and sustainability [48,49]. Higher polyphenol extraction yields obtained using ethanol–water binary solvent systems have been widely documented. This effect is explained by ethanol’s ability to improve polyphenol solubility, as it reduces the dielectric constant of the aqueous medium and facilitates their diffusion throughout the solvent [50]. At the same time, water facilitates the desorption of these compounds from the plant matrix, thereby further improving extraction efficiency [51].

Regarding the influence of ethanol concentration, Figure 1c shows that at low ultrasonic power, increasing the ethanol concentration from 40% to 80% results in a higher yield of phenolic compounds in the extract. However, at power values above 100 W, the opposite phenomenon was observed, with extraction being favored at lower ethanol concentrations. The decrease in polyphenol extraction yield upon increasing ethanol concentration to 80–90% has often been reported in previous studies. Cacace and Mazza [52] observed that total phenolic extraction from black currants peaked at roughly 60% ethanol and declined at higher concentrations. Yılmaz et al. [53] found that the maximum predicted total phenolic content in the extracts from sour cherry pomace was obtained at 55% EtOH and decreased above this concentration while Drevelegka and Goula [54] reported that the highest UAE yields of phenolics from grape pomace was achieved with 50% ethanol. Bamba et al. [55] also reported that increasing the ethanol concentration from 50% to 90% led to significantly lower total phenolic content (TPC) and DPPH free-radical scavenging activity in the extracts. They attributed this effect to ethanol’s dehydrating action on plant cells, which may hinder the diffusion of polyphenols from the plant material into the solvent. Wang et al. [56] similarly demonstrated a concentration-dependent response in phenolic recovery from blueberry leaves, where increasing ethanol levels from 40% to 70% enhanced extraction efficiency, whereas a further rise to 90% ethanol resulted in a noticeable decline in yield, suggesting the presence of an optimal solvent composition for maximum recovery. These differences arise from both the matrix and phenolic composition, as plant extraction involves three main steps: solvent diffusion into the plant matrix, desorption of target compounds due to solvent affinity, and mass transfer of solutes into the bulk solvent [57]. Polyphenols are highly diverse in their functional groups; thus, compounds such as flavonoid aglycones, phenolic terpenes, and methoxylated phenolic acids require less polar solvents for extraction. Highly polar solvents, including ethanol and water, are more effective at dissolving polar polyphenolic compounds due to their stronger molecular interactions, thereby enhancing the overall efficiency of their extraction [58]. For instance, quercetin shows high solubility in alcohols, with increased yields at ≥70% ethanol [59]. In addition to solvent solubility, mass transport phenomena arising from interactions between the plant matrix, solvent, and target compounds must be considered during extraction. The solvent affects cellular permeability by inducing chemical or biophysical changes; for example, ethanol increases permeability by interacting with the phospholipid bilayer [59], while water promotes polyphenol diffusion from plant cells by inducing tissue swelling, which aids their release [60].

Extending the extraction duration from 30 min to 60–70 min led to a progressive enhancement of polyphenol yield. However, extending the process beyond this optimal range resulted in a gradual decline in yield; by 90 min, the recovered polyphenol content falls to levels similar to those observed at 30 min. This pattern indicates the presence of a time-dependent optimum, followed by reduced extraction efficiency under prolonged processing conditions.

### 2.4. Effect of UAE on DPPH Radical Scavenging Activity

**Figure 3:**
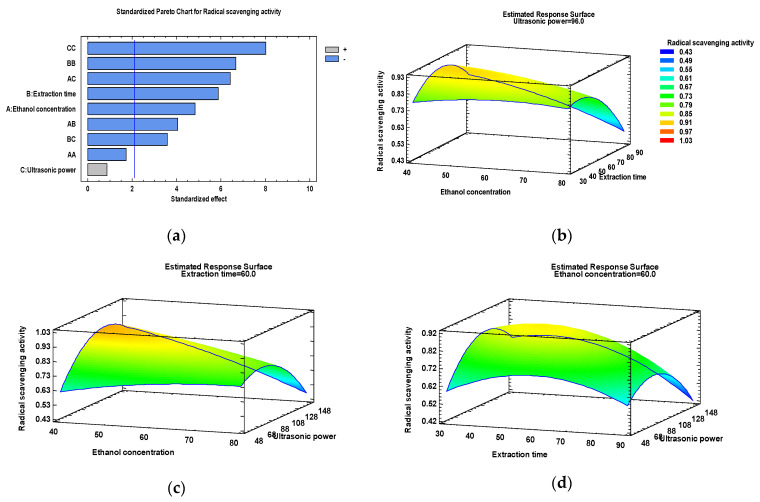
Pareto chart (**a**) and estimated response surface graphs of DPPH radical scavenging activity (mmol Trolox/L) as a function of ethanol concentration and extraction time at 96 W ultrasonic power (**b**), ethanol concentration and ultrasonic power at 60 min extraction time (**c**) and extraction time and ultrasonic power at 60% ethanol concentration (**d**).

Figure 3b–d displays the response surface plots illustrating the interactive effects of two process variables on RSA. In each case, the third parameter was fixed at its central level to enable visualization of the corresponding two-factor interactions. Based on the model, RSA of the pear pomace extract records the maximum value (0.93 mmol Trolox/L) using 40% ethanol as a solvent, after 53.31 min extraction time at 117.44 ultrasonic power. The R-squared value indicates that the fitted model accounts for 92.42% of the variability observed in RSA. As presented in Figure 3a, RSA was significantly influenced by the linear, quadratic and combined effect of all process parameters (*p* < 0.05) except the linear effect of ultrasonic power and the quadratic effect of ethanol concentration.

When evaluating the effects of ethanol concentration and extraction time (Figure 3b), it was noticed a higher analytical response at lower ethanol concentration, mostly as the extraction time extended beyond 30 min. Similar to the trend observed for TPC, RSA increased with increasing ultrasonic power up to an optimum point, after which it declined (Figure 3c,d).

A statistically significant positive correlation was found between TPC and RSA in our study, with a coefficient of correlation of 0.727, indicating a moderately strong relationship between these two variables. A positive correlation between RSA and TPC in pear pomace has also been reported by other authors [10].

### 2.5. Phenolic Characterization of Pear Pomace Extracts

Table 5 presents the content of phenolic compounds (mg/L), as quantified by HPLC, in alcoholic pear pomace extracts obtained in selected UAE conditions at the central point of the experimental design for ultrasonic power (96 W). Chromatograms of phenolic compounds at 254, 278 and 300 nm of the pear pomace extract (run 5 in the Box–Behnken experimental design) are shown in Figure S1a, b and c, respectively, available in the “Supplementary Material”.

Chlorogenic acid was found to be the dominant compound within the profile, followed by rutin and epicatechin, which were present in comparatively lower but still notable amounts. Krajewska et al. [10] found also chlorogenic acid as the dominant polyphenolic acid in pear pomace while Wang et al. [56] identified chlorogenic acid, vanillic acid, ferulic acid, and rutin as the primary bioactive constituents in Yaguang pear (*P. ussuriensis* Maxim) peel extract. Ferreira et al. [8] similarly identified chlorogenic acid as the most prevalent phenolic compound in pear pomace powder, reporting concentrations between 15.3 and 23.6 mg/100 g dw. Krajewska et al. [10] also reported that pear pomace contained particularly high levels of rutin, ranging from 0.92 to 2.67 mg/g dw, and epicatechin ranging from 13.78 to 21.59 mg/g dw. They demonstrated that drying method as well as drying temperature generally affected the content of individual phenolic compounds.

Earlier studies have emphasized the high antioxidant activity of chlorogenic acid and its major contribution to the health benefits associated with pear consumption [8]. The high content of phenolic compounds in pear pomace extracts further supports their potential use as a functional food ingredient for improving dietary antioxidant intake, or as components of dietary supplements.

The results presented in Table 5 demonstrate that the extraction parameters (ethanol concentration and extraction time) significantly affected the extraction yield of individual phenolic compounds. Extraction yield increased as the extraction time was extended from 30 to 60 min; however, a further increase to 90 min resulted in a decrease in the yield of most individual phenolic compounds, irrespective of the ethanol concentration in the solvent.

Extraction in 40% ethanol for 60 min was the most efficient for most phenolic compounds. However, the highest extraction efficiency of rutin was obtained using 60% ethanol for 60 min, as was the case for gallic acid. Good extraction yields were also obtained using 80% ethanol for rutin, catechin-hydrate, epicatechin, syringic, trans-cinnamic and chlorogenic acids. This differential behavior may be attributed to the polarity of the solvent and the differential solubility of polyphenolic compounds [61]. Ethanol has lower polarity than water; consequently, hydroethanolic mixtures become more polar as the proportion of ethanol decreases. Previous studies reported that solubility of phenolic compounds in aqueous ethanol is based on the “polarity versus polarity” principle with ethanol playing a crucial role in disrupting the hydrogen and hydrophobic interactions between phenolics and proteins, as well as between phenolics and cellulose, within the water–ethanol system [54]. The chemical structure of phenolic compounds, including the degree of conjugation and the number of hydroxyl groups, plays a key role in determining their solubility in different solvents. Moreover, a higher water content in the solvent can enhance the extraction of phenolic glycosides [62].

Based on Pearson correlation analysis, the only phenolic compounds whose contents exhibited significant (*p* < 0.05) positive linear correlations with DPPH antioxidant activity were ferulic (r = 0.97, R^2^ = 94.91%), p-coumaric (r = 0.96, R^2^ = 93.26%), chlorogenic (r = 0.76, R^2^ = 78.07%), and trans-cinnamic acids (r = 0.76, R^2^ = 77.42%). These findings could be attributed to the antioxidant activity of individual phenolic compounds, which is strongly dependent on the number and position of their available hydroxyl groups, and to the chemical interactions—such as synergistic, antagonistic, and additive effects—occurring among various phenolic compounds, as previously reported in many other complex food matrices [10,63,64].

## 3. Materials and Methods

### 3.1. Materials

Pears (*Pyrus communis* L.), cultivar “Abate Fetel” were collected at commercial maturity from an orchard located in Dolj County, Romania, during the 2025 harvest season. After refrigerated storage (4 °C) for 2 weeks, the fruits were processed to obtain juice in a juice extractor (R.G.V., Como, Italy). Fresh pomace samples, composed of peel, core, seeds, and pulp fragments, were collected directly after juice extraction and homogenized for 2 min using a laboratory blender (Tefal Smart, MB450141, Istanbul, Turkey). The resulting by-product was evenly distributed in a thin layer (2 mm) on parchment paper and dried at 63 °C to a final moisture content of 7–8% using a forced-convection hot air dryer (Deca +SS Design, Profimatic, Cluj-Napoca, Romania). The dried pomace was ground using a household electric grinder, passed through a 0.5 mm mesh, packed in bags and stored in the dark at room temperature (20 °C) until analyses and extraction experiments.

### 3.2. Chemicals

Folin–Ciocalteu reagent (2 N), 2,2-diphenyl-1-picrylhydrazyl (DPPH), 6-hydroxy-2,5,7,8-tetramethylchroman-2-carboxylic acid (Trolox), anhydrous sodium carbonate, acetic acid and ethanol were of analytical grade and were purchased from Sigma-Aldrich (Steinheim, Germany). Standards of phenolic acids (gallic, caffeic, chlorogenic, *p*-coumaric, ferulic, syringic, *trans*-cinnamic and vanillic) and flavonoids (quercetin, catechin-hydrate, epicatechin and rutin) were also obtained from Sigma-Aldrich GmbH (Steinheim, Germany). Methanol (HPLC grade) used in the chromatographic analysis was from Merk (Darmstadt, Germany).

### 3.3. Box–Behnken Experimental Design and Optimization by RSM

Response surface methodology (RSM) was employed to optimize the ultrasonic-assisted extraction (UAE) of phenolic compounds from pear pomace in aqueous ethanol. Various process parameters, such as solvent type, temperature, extraction time, solvent-to-material ratio, and pH, are known to influence the extraction yield of phenolic compounds [65]. In the present study, a three factor Box–Behnken design was employed to model the influence of ethanol concentration (40 to 80%), extraction time (30 to 90 min) and ultrasonic power (48 to 144 W) on total phenolic content (TPC) and DPPH radical scavenging activity (RSA) of pear pomace extracts. The experimental design was conducted at three levels (−1, 0, and +1), comprising 15 runs performed in duplicate, for a total of 30 experimental runs. The independent variables and their levels are shown in Table 6.

The behavior of the model is explained by the following second-order nonlinear quadratic polynomial equation:

$$Y=\beta_{0}+\sum_{i=1}^{k}\beta_{i}x_{i}+\sum_{i=1}^{k}\beta_{ii}x_{i}^{2}+\sum_{i=1}^{k-1}\sum_{j=2}^{k}\beta_{ij}x_{i}x_{j}$$

where Y is the process response (dependent variable), k is the number of the tested factors (k = 3 in this study), i and j are the index numbers for tested factors, β_0_ is the free term (intercept term), x_1_, x_2_,…, x_k_ are the independent variables, β_i_ are the first-order (linear) terms, β_ii_ are the quadratic (squared) terms, β_ij_ are the interaction terms of variables i and j, respectively. The appropriate ranges for the extraction factors were selected based on information from the literature and preliminary experiments. Experimental data were analyzed using Statgraphics Centurion XVI software (StatPoint Technologies, Warrenton, VA, USA). ANOVA was applied to evaluate model adequacy, and R-Squared (*R*^2^) was used to measure the agreement between predicted and observed values. Model significance was established at the 95% confidence level (*p* < 0.05). Response surface graphs were plotted to evaluate the relationship between two independent variables and a response.

### 3.4. Extraction Procedure

For each run, a sample of powdered pear pomace (1.5 g) was mixed with 30 mL solvent (40, 60 and 80% aqueous ethanol) in a 50 mL flask resulting in a solid/solvent ratio of 5%. Extractions were performed in a Bandelin Sonorex Digital 10P ultrasonic bath (Bandelin Electronic GmbH, Berlin, Germany) operating at 35 kHz. Ultrasonic power was adjusted at 48, 96 and 144 W (corresponding to 10%, 20% and 30% of the maximum ultrasonic power), according to the experimental design presented in Table 2. The ultrasonic bath was operated using water at 25 °C and, during extraction, the temperature was continuously monitored and maintained at the target level (±1 °C) by periodically adding cold water (5 °C). After the extraction time was completed (30, 60 and 90 min, based on the combinations of extraction parameters described in Table 2), the extracts were centrifuged at 6000 rpm for 10 min and the supernatants were collected, filtered through Whatman filter paper (8–12 μm pore size) and preserved at −20 °C until analysis.

### 3.5. Total Phenolic Content

Total phenolic content was quantified by the spectrophotometric Folin–Ciocalteu assay, employing gallic acid as the reference standard, following the procedure of Singleton et al. [66]. Briefly, a mixture containing 0.1 mL of extract, 5 mL of distilled water, and 0.5 mL of freshly diluted (1:1) Folin–Ciocalteu reagent was combined with 1.5 mL of 20% Na_2_CO_3_ and 2.9 mL of distilled water. After 30 min in the dark at 40 °C, absorbance was read at 765 nm using a Varian Cary 50 UV spectrophotometer (Varian Co., Palo Alto, CA, USA), and results were expressed as mg GAE/L of extract.

### 3.6. DPPH Radical Scavenging Activity

DPPH radical scavenging activity was assessed following the method of Brand-Williams et al. [67]. The assay was performed by mixing 50 μL of pear pomace extract with 3 mL of a 0.004% DPPH methanolic solution. Following shaking, the mixture was incubated in the dark for 30 min at 20 °C, and absorbance was subsequently recorded at 517 nm against a methanol blank. A control, prepared with methanol instead the pomace extract, was used to measure the maximum DPPH absorbance. DPPH scavenging activity was calculated as percent inhibition using the formula:

DPPH scavenging activity (%) = [1 − absorbance of the sample/absorbance of the control] × 100

A Trolox calibration curve was constructed and results were expressed as mmol Trolox/L of extract.

### 3.7. Quantification of Individual Polyphenolic Compounds

Quantification of twelve phenolic compounds in the pear pomace extracts was performed by HPLC-DAD, following the method of Nour et al. [68], using a Finnigan Surveyor Plus HPLC equipped with a diode-array detector (Thermo Electron Corporation, San Jose, CA, USA). Chromatographic separation was performed at 20 °C on a Hypersil Gold C18 column (5 µm, 250 × 4.6 mm). The mobile phase, consisting of 1% aqueous acetic acid (A) and methanol (B), was eluted at a flow rate of 1 mL/min according to the following gradient elution program: 0–20 min (from 90% A to 80% A), 20–27 min (from 80% A to 60% A), 27–52 min (60% A), 52–57 min (from 60% A to 80% A) and 57–60 min (from 80% A to 90% A). Chromatograms were acquired simultaneously at 254, 278, and 300 nm. A 5 µL aliquot of the pear pomace extract was injected after filtration through a 0.45 µm nylon syringe filter. Analyte peaks were confirmed by matching their retention times and UV spectra to those of reference standards. Phenolic compound concentrations were calculated from peak areas using external calibration and reported as mg/L of extract.

### 3.8. Statistical Analysis

Response surface methodology (RSM) was conducted using Statgraphics Centurion XVI.I software (StatPoint Technologies, Warrenton, VA, USA). All analyses were performed in triplicate, and the results are presented as mean ± standard deviation (SD). Data were statistically evaluated using one-way analysis of variance (ANOVA) followed by the least significant difference (LSD) test, with statistical significance set at *p* < 0.05.

## 4. Conclusions

Pear pomace is a sustainable and abundant source of phenolic compounds with strong antioxidant potential, which can be efficiently recovered through ultrasound-assisted extraction using aqueous ethanol as the solvent. The process was optimized via response surface methodology considering ethanol concentration (40–80%), extraction time (30–90 min) and ultrasonic power (48–144 W) as independent variables and total phenolic content and DPPH radical scavenging activity as response variables. Optimal extraction parameters (40% ethanol concentration, 117.6 W ultrasonic power intensity, and 64.45 min extraction time) enabled the highest total phenolic recovery (280.1 mg GAE/L). The phenolic profile of the extracts was dominated by chlorogenic acid, followed by rutin and epicatechin. Extraction time and ethanol concentration significantly influenced the yield of individual phenolic compounds. This study supports the valorization of pear pomace via an environmentally friendly extraction approach, highlighting its potential as a source of natural antioxidants. Future research should focus on evaluating the economic viability of the recovery process, as well as on exploring the potential applications of the obtained extracts in the food and pharmaceutical industries.

## Figures and Tables

**Table 1:** Proximate composition, titratable acidity, total phenolic content and DPPH radical scavenging activity of pear pomace powder.

Dry Matter (%)	Protein (%)		Fat (%)	Cellulose (%)		Ash (%)
91.98 ± 0.57	3.84 ± 0.19		0.77 ± 0.04	12.21 ± 0.36		1.52 ± 0.07
**Titratable Acidity** **(g Malic Acid/100 g)**		**Total Phenolic Content** **(mg GAE/100 g)**			**DPPH Radical Scavenging** **Activity (mmol Trolox/100 g)**	
1.03 ± 0.08		297.98 ± 11.10			0.92 ± 0.07	

**Table 2:** Phenolic content (mg/100 g) in pear pomace powder.

Vanillic Acid	Rutin	Quercetin	Gallic Acid	Catechin Hydrate	Syringic Acid
3.61 ± 0.18	18.32 ± 0.16	0.13 ± 0.01	0.19 ± 0.02	0.92 ± 0.08	0.02 ± 0.01
**Epicatechin**	***Trans*** **-** **Cinnamic Acid**	**Chlorogenic Acid**	**Caffeic Acid**	***p*** **-Coumaric Acid**	**Ferulic Acid**
6.26 ± 0.08	nd	26.41 ± 0.67	0.16 ± 0.01	0.14 ± 0.01	0.56 ± 0.02

**Table 3:** Factorial design and response values for total phenolic content and DPPH radical scavenging activity in the ultrasonic-assisted extraction of phenolic compounds from pear pomace.

	Process Parameters (Actual Values)			Total Phenolic Content (mg GAE/L)		DPPH Radical Scavenging Activity (mmol Trolox/L)	
Design Point	Ethanol Concentration (%)	Extraction Time (min)	Ultrasonic Power (W)	Observed	Predicted	Observed	Predicted
1	60.0	60.0	96.0	257.45	263.62	0.86	0.88
				262.91	260.62	0.84	0.86
2	60.0	60.0	96.0	267.45	263.62	0.86	0.88
				257.45	260.62	0.90	0.86
3	80.0	90.0	96.0	251.09	247.71	0.44	0.50
				256.55	244.70	0.43	0.49
4	60.0	30.0	48.0	160.18	159.64	0.53	0.60
				152.91	156.63	0.55	0.58
5	80.0	60.0	144.0	180.18	192.71	0.51	0.52
				183.82	189.70	0.54	0.50
6	40.0	30.0	96.0	251.09	257.48	0.85	0.78
				245.64	254.48	0.82	0.77
7	80.0	60.0	48.0	235.64	240.89	0.78	0.74
				236.55	237.89	0.80	0.72
8	60.0	90.0	144.0	168.36	162.82	0.54	0.47
				157.45	159.81	0.47	0.45
9	40.0	90.0	96.0	252.02	258.39	0.79	0.78
				252.04	255.38	0.78	0.76
10	40.0	60.0	144.0	257.45	253.84	0.83	0.89
				253.82	250.83	0.80	0.87
11	80.0	30.0	96.0	262.00	258.16	0.79	0.81
				261.09	255.16	0.78	0.79
12	60.0	90.0	48.0	192.06	195.09	0.58	0.58
				186.55	192.10	0.55	0.56
13	60.0	30.0	144.0	212.91	207.82	0.78	0.75
				208.36	204.82	0.71	0.73
14	40.0	60.0	48.0	199.91	189.75	0.65	0.63
				195.03	186.75	0.56	0.61
15	60.0	60.0	96.0	267.45	263.62	0.88	0.88
				260.03	260.62	0.89	0.86

**Table 4:** Regression coefficients and analysis of variance (ANOVA) for the quadratic models predicting TPC and RSA.

Regression Coefficients	Total Phenolic Content	DPPH Radical Scavenging Activity
*β*_0_	−194.621 (0.2707)	−1.66687 (0.3939)
*β*_1_ (ethanol concentration)	−1.49966 (0.1840)	0.0262187 * (0.0001)
*β*_2_ (extraction time)	4.6216 (0.2038)	0.0264653 * (0.0000)
*β*_3_ (ultrasonic power)	7.60919 * (0.0410)	0.0232986 (0.4005)
*β*_12_	−0.00473333 (0.2818)	−0.000125 * (0.0007)
*β*_13_	−0.0292383 * (0.0000)	−0.000123698 * (0.0000)
*β*_23_	−0.0139687 * (0.0000)	−0.0000460069 * (0.0020)
*β*_11_	0.037213 * (0.0000)	−0.0000833333 (0.0996)
*β*_22_	−0.0256345 * (0.0000)	−0.000142593 * (0.0000)
*β*_33_	−0.0256976 * (0.0000)	−0.0000670935 * (0.0000)
*p*-value	0.0000	0.0000
*R* ^2^	97.6638	92.4161

* Significance (*p* ˂ 0.05); *p*-values in parentheses.

**Table 5:** Phenolic compounds (mg/L) determined by HPLC in the alcoholic pear pomace extracts obtained in selected UAE conditions (ultrasonic power = 96 W).

Ethanol Concentration (%)	Extraction Time (min)	Vanillic Acid	Rutin	Quercetin	Gallic Acid	Catechin Hydrate	Syringic Acid
40	30	1.16 ± 0.02 ^de^	8.41 ± 0.28 ^e^	0.15 ± 0.01 ^f^	0.04 ± 0.01 ^de^	1.68 ± 0.05 ^a^	0.14 ± 0.01 ^f^
40	60	2.57 ± 0.11 ^a^	9.84 ± 0.32 ^c^	1.44 ± 0.04 ^a^	0.08 ± 0.01 ^b^	1.70 ± 0.06 ^a^	0.37 ± 0.02 ^a^
40	90	2.14 ± 0.08 ^b^	7.46 ± 0.26 ^f^	0.19 ± 0.01 ^e^	0.05 ± 0.01 ^cd^	1.40 ± 0.03 ^e^	0.29 ± 0.02 ^cd^
60	30	1.17 ± 0.02 ^de^	9.95 ± 0.32 ^c^	0.15 ± 0.00 ^f^	0.05 ± 0.02 ^cd^	1.21 ± 0.04 ^c^	0.15 ± 0.01 ^f^
60	60	1.22 ± 0.01 ^de^	11.75 ± 0.36 ^a^	0.28 ± 0.02 ^d^	0.13 ± 0.01 ^a^	1.38 ± 0.03 ^b^	0.25 ± 0.01 ^d^
60	90	1.15 ± 0.04 ^e^	10.26 ± 0.25 ^c^	0.18 ± 0.01 ^ef^	0.06 ± 0.01 ^c^	1.08 ± 0.02 ^d^	0.18 ± 0.01 ^e^
80	30	1.25 ± 0.09 ^d^	9.12 ± 0.27 ^d^	0.26 ± 0.02 ^d^	0.06 ± 0.00 ^c^	1.37 ± 0.06 ^b^	0.31 ± 0.02 ^c^
80	60	1.37 ± 0.01 ^c^	11.08 ± 0.44 ^b^	0.33 ± 0.02 ^c^	0.08 ± 0.01 ^b^	1.67 ± 0.07 ^a^	0.34 ± 0.02 ^b^
80	90	0.85 ± 0.01 ^f^	8.81 ± 0.21 ^de^	0.43 ± 0.03 ^b^	0.03 ± 0.01 ^e^	1.17 ± 0.04 ^c^	0.18 ± 0.02 ^e^
		**Epicatechin**	***Trans*** **-** **Cinnamic Acid**	**Chlorogenic Acid**	**Caffeic** **Acid**	***p*** **-Coumaric Acid**	**Ferulic** **Acid**
40	30	5.52 ± 0.13 ^c^	1.60 ± 0.05 ^c^	13.96 ± 0.57 ^c^	0.06 ± 0.01 ^b^	0.11 ± 0.01 ^b^	0.25 ± 0.02 ^a^
40	60	6.26 ± 0.24 ^a^	1.96 ± 0.07 ^a^	16.12 ± 0.49 ^a^	0.12 ± 0.01 ^a^	0.14 ± 0.01 ^a^	0.22 ± 0.01 ^a^
40	90	6.36 ± 0.28 ^a^	1.60 ± 0.05 ^c^	14.02 ± 0.45 ^c^	0.06 ± 0.01 ^b^	0.11 ± 0.01 ^b^	0.22 ± 0.02 ^a^
60	30	3.45 ± 0.12 ^e^	1.42 ± 0.06 ^d^	12.36 ± 0.37 ^d^	0.06 ± 0.02 ^b^	0.13 ± 0.02 ^ab^	0.26 ± 0.05 ^a^
60	60	4.85 ± 0.11 ^d^	1.75 ± 0.05 ^b^	15.23 ± 0.40 ^b^	0.09 ± 0.01 ^ab^	0.14 ± 0.01 ^a^	0.26 ± 0.04 ^a^
60	90	3.28 ± 0.08 ^e^	1.34 ± 0.00 ^d^	11.78 ± 0.31 ^d^	0.05 ± 0.01 ^b^	0.11 ± 0.03 ^b^	0.24 ± 0.01 ^a^
80	30	5.54 ± 0.19 ^c^	1.88 ± 0.06 ^a^	15.99 ± 0.47 ^ab^	0.08 ± 0.01 ^ab^	0.08 ± 0.01 ^c^	0.24 ± 0.01 ^a^
80	60	5.87 ± 0.22 ^b^	1.55 ± 0.06 ^c^	15.66 ± 0.54 ^ab^	0.08 ± 0.01 ^ab^	0.12 ± 0.01 ^ab^	0.26 ± 0.01 ^a^
80	90	4.81 ± 0.11 ^d^	1.34 ± 0.05 ^d^	12.17 ± 0.33 ^d^	0.06 ± 0.01 ^b^	0.07 ± 0.01 ^c^	0.16 ± 0.01 ^b^

Different lowercase letters within each column indicate statistically significant differences between samples (*p* < 0.05).

**Table 6:** Coded and actual values of the independent variables used in the Box–Behnken design.

		Levels		
Symbols	Independent Variables	Low Level (−1)	Center Level (0)	High Level (+1)
		Actual Values		
x_1_	Ethanol concentration (%)	40	60	80
x_2_	Extraction time (min)	30	60	90
x_3_	Ultrasonic power (W)	48	96	144

## Data Availability

The original contributions presented in this study are included in the article. Further inquiries can be directed to the corresponding author.

## Supplementary Materials

The following supporting information can be downloaded at: https://www.mdpi.com/article/10.3390/molecules31111938/s1, Figure S1: Chromatograms of phenolic compounds at (a) 254, (b) 278 and (c) 300 nm of the pear pomace extract (run 5 in the Box–Behnken experimental design).

## Abbreviations

The following abbreviations are used in this manuscript:

RSM	Response Surface Methodology
UAE	Ultrasound-assisted extraction
TPC	Total phenolic content
RSA	Radical scavenging activity
HPLC-DAD	High-performance liquid chromatography with diode-array detection
