# Supplementary material for: Ultrasound-Assisted Extraction of Phenolics from Pear Pomace: Method Optimization, Phenolic Profile, and Antioxidant Capacity
Source: Molecules. 2026 Jun 3;31(11):1938. doi: 10.3390/molecules31111938 (PMC13258349; doi:10.3390/molecules31111938)
Supplement: Supplementary file 1 [file molecules-31-01938-s001.zip › molecules-4297089-supplementary.pdf]

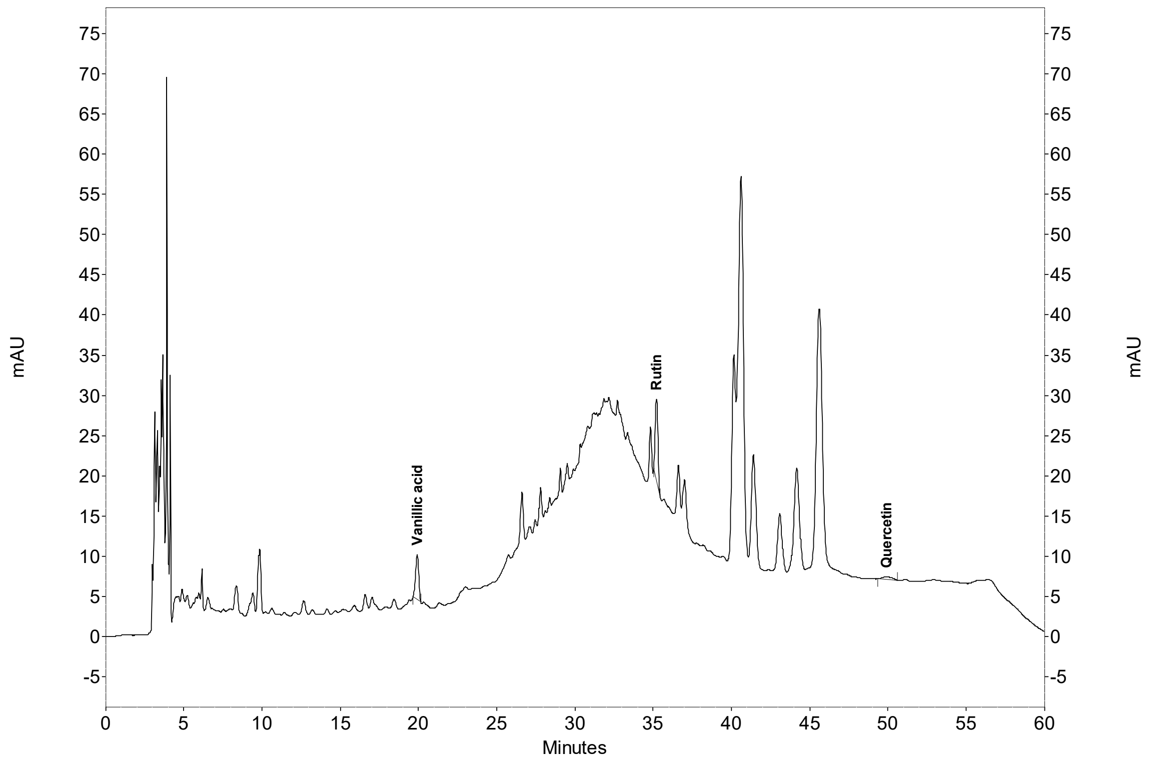

Vanillic acid
Rutin
Quercetin
mAU
Minutes

(a)

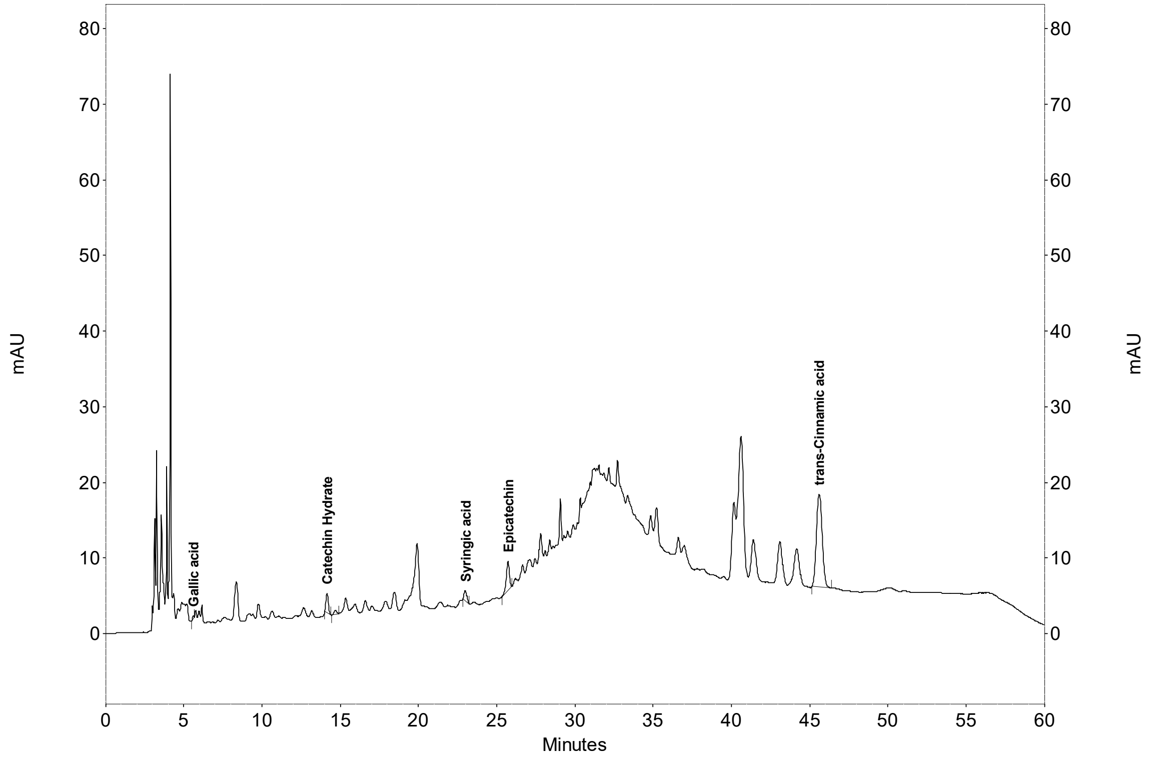

Gallic acid
Catechin Hydrate
Syringic acid
Epicatechin
trans-Cinnamic acid
mAU
Minutes

(b)

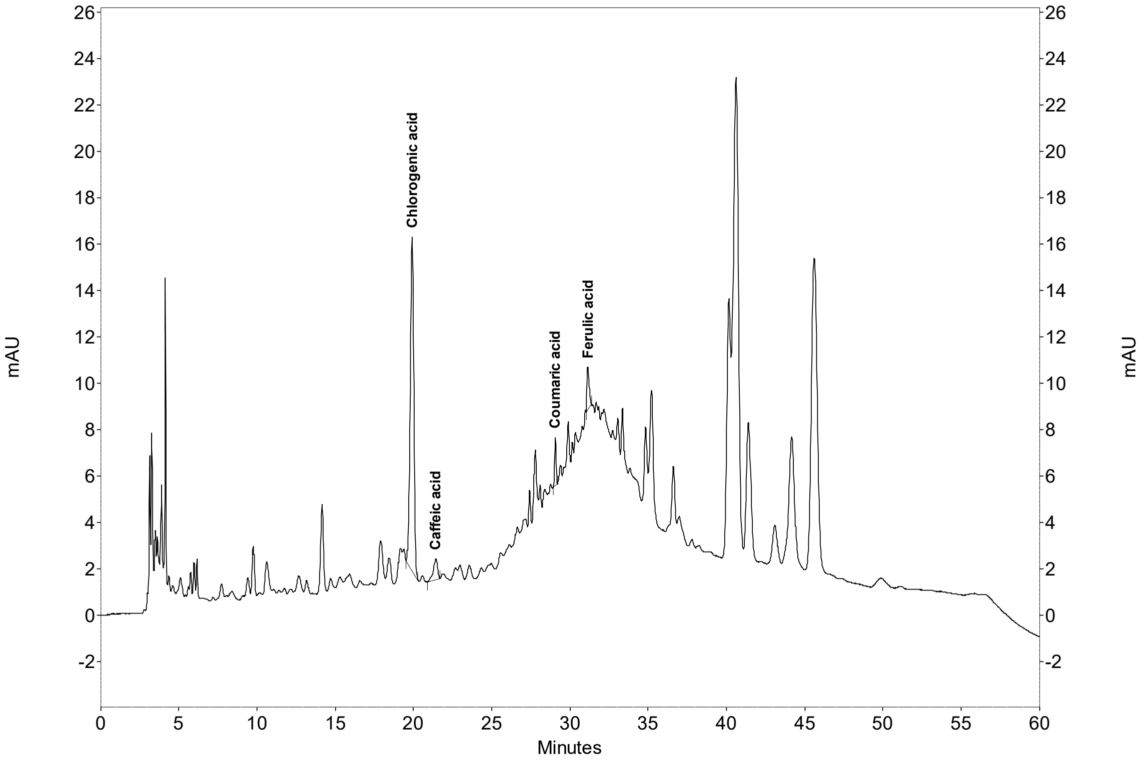

(c)

Figure S1. Chromatograms of phenolic compounds at (a) 254, (b) 278 and (c) 300 nm of the pear pomace extract (run 5 in the Box–Behnken experimental design).
